# The role of large language models in the writing of surgical reviews: fact or fantasy?

**DOI:** 10.1007/s00423-026-04095-2

**Published:** 2026-07-03

**Authors:** Daphne E. DeTemple, Simon Störzer, Sahar Arbabzadah, Anna Riddermann, Felix Gronau, Kai Timrott, Hüseyin Bektas, Moritz Kleine

**Affiliations:** 1 Department for General-, Visceral- and Transplant Surgery, Hannover Medical School, Hannover, Germany; 2 Department for General-, Visceral-, Pediatric- and Transplant Surgery, University Clinic RWTH Aachen, Aachen, Germany; 3 PRACTIS Clinician Scientist Program, Dean’s Office for Academic Career Development, Hannover Medical School, Hannover, Germany; 4 Clinic for General- and Visceral Surgery, Agaplesion Ev. Klinikum Schaumburg, Obernkirchen, Germany; 5 Clinic for General-, Visceral- and Oncological Surgery, Klinikum Bremen Mitte, Bremen, Germany; 6 Clinic for General-, Visceral- and Oncological Surgery and Coloproctology, Vinzenzkrankenhaus Hannover, Hannover, Germany

**Keywords:** AI-text generation, Scientific writing, Hepatobiliary surgery, Readability, Large language model

## Abstract

**Background:**

Amidst the current enthusiasm concerning artificial intelligence and its possible application in the composition of different kinds of scientific and non-scientific written documents, we evaluated the usage of artificial intelligence for writing surgical short reviews.

**Methods:**

In order to assess the formal and content quality of AI-generated texts compared to human written texts, ten AI-based text generators (five chatbots and five content creators) and four surgeons in training received the same prompt for a short scientific article on a liver surgery theme. All texts were anonymized and subsequently evaluated by three experienced liver surgeons based on a pre-defined scoring scheme, as well as for quality of references and readability according to readability indices. Furthermore, all texts were tested for plagiarism using PlagScan.

**Results:**

Overall percentage of correct assessment for AI/non-AI generation by experienced surgeons lay at 78.57%. Human written text had a mean word count of 1054 versus 874 in AI-generated text, with a higher mean Flesh Reading Ease Score (FRE, 26.2 ± 5.1 versus 17.7 ± 6.1). References were PubMed-listed in 100% for human versus 46% for AI-generated text, with only one non-human text reaching 100% formally correct citation of references. PlagScan found 6.4%±1.3 mean resemblance to existing texts for human versus 7.6%±4.5 for AI-generated text.

**Discussion:**

Overall, AI could already mislead experienced scientific surgeons in 26.7% of cases into believing it to be human. However, formal requirements, especially considering referencing, are still in great need of improvement with only one of AI-generated articles fulfilling our quality requirements.

## Introduction

Artificial intelligence (AI)-based large language models (LLM) in the form of chatbots have changed not only the way the general population performs internet searches, but also the scientific writing community. Since its release for general use in November 2022, especially OpenAI’s ChatGPT, but also other providers, such as Google’s Bard, or independent hosts, have gained not only much attention, but also influence on academic writing in schools, universities and research. In medicine, patient communication as well as clinical documentation have also come into focus as possible applications for AI-based LLMs [1]. Before going into detail concerning the application of AI in the writing of scientific, surgical texts in the example of a short review, we will give a short overview on the current role of AI in medical academia.

When considering employing AI in the writing of texts of any kind, the language level, as well as comprehensibility, validity and quality of writing have to be taken into account. In general, AI-based LLMs are considered to have the ability to generate texts on basically any topic at the level of high school to college students. Due to the discussion on the recognizability of AI-generated versus human-written texts, several authors have discussed the need for an update of ethics and regulations for the written word in order to minimize incorrect content and consecutive loss of trust [2, 3] resulting from the use of AI.

Concerning medical applications, pathology and radiology working with large amounts of imaging data, which need to be analyzed based on specific patterns signifying different pathologies to find diagnoses, seem the most obvious option. Accordingly, papers on the use especially in radiology as most digital medical specialty, have already reached over 260 since the release of ChatGPT when prompting PubMed with the key words “radiology” and “large language model”. The general agreement seems to be that the fast analysis of large amounts of data in the internet may be used not only in the training of medical students and doctors [4], but also for patient communication or even as support for medical decision making [5]. Some authors have already addressed a possible application for scientific medical writing: Whereas most see the chances of LLMs to facilitate literature reviews or summaries [6], some concentrate more on possible ethical concerns [7] prompting high-ranked journals like Nature to release rules on the use of LLMs in scientific writing [8].

When looking into applications in scientific surgical writing, literature is not as abundant. Whilst some esthetic and plastic surgeons appear confident on a broad applicability for research on their field, e.g. for the generation of review ideas, even though accuracy of answers given by ChatGPT ranged from 35 to 75% [9, 10], others see the limitations concerning data privacy and need for human input [11].

One recent Chinese study [12] evaluated the possibility to let ChatGPT write an article section complete with references on different fields, which they then evaluated for accuracy of the digital object identifiers (DOI) given. Though published in April 2024, the researchers had used GPT-3.5 and found that the existence of given DOIs was relatively low (38 and 71%, depending on field of research) with up to 89% “hallucination” of DOIs.

In this project, seven different AI platforms were used for generation of ten texts as shown in Table 1. These include OpenAI’s ChatGPT in two different versions (GPT-3.5 and GPT-4plus), the chat-based assistant Perplexity, the AI content generation platforms AI-Writer, Neuroflash and Notion as well as the chat-based assistants and content creation platforms by Writesonic and Japsper. As Google’s Bard was not yet published at the time of article generation, it was not included in this analysis.

**Table 1:** Overview on included AI-tools with underlying LLM, categories: chat-based assistant (CBA) or content creation platform (CCP), release date and training type [13–19]

	LLM	Category	Release	Type of training
ChatGPT	GPT (OpenAI)	CBA	2022	large-scale pretraining on unspecified diverse text data, supervised fine-tuning and reinforcement learning from human feedback (RLHF)
AI-Writer	Palmyra (AI Writer Technologies)	CCP	2020	Unclear, in part scientific texts
Perplexity	Qwen (Alibaba)	CBA	2023	trained on large-scale multilingual web, code, and academic data, supervised fine-tuning and reinforcement learning from human feedback (RLHF)
Writesonic, Chatsonic	multi-modal (GPT, Claude, Gemini)	CBA	2022	No individual training by the platform; use of existing LLMs
Writesonic, AI Article Writer	GPT	CCP	2021	No individual training by the platform; use of OpenAI’s GPT
Neuroflash	GPT	CCP	2021	No individual training by the platform; use of OpenAI’s GPT
JasperChat	GPT	CBA	2023	No individual training by the platform; use of OpenAI’s GPT
Jasper Document editor	GPT	CCP	2022	No individual training by the platform; use of OpenAI’s GPT
Notion	GPT	CCP	2023	No individual training by the platform; use of OpenAI’s GPT

## Materials and methods

### Study design

Ten article generators and chatbots based on artificial intelligence (AI) as well as four surgeons in training were given the same prompt to write a short scientific review. None of the surgeons in training were native English speaking and only two had previously written scientific text in the form of a doctoral thesis.

The prompt given to all human and AI-writers was: “Write a short scientific review on ‘Laparoscopic versus robotic liver surgery – benefits and challenges depending on the indication’. Give PubMed-listed references in AMA-style”. All human and AI authors were limited to three pages or 1 500 words. The AI chatbots and article generators included were: ChatGPT (versions 3.5 and 4.0), Writesonic (Chatsonic and AI Article Writer), AI-Writer, Perplexity, Jasper (Chat and Document editor), Neuroflash and Notion. An overview on the underlying LLMs as well as training strategies is given in Table 1. All texts were copied into Microsoft Word and saved as pdf with random numbers assigned to each text to ensure anonymization. All texts were subsequently evaluated according to the score sheet given in Table 2 by three experienced hepatobiliary surgeons. These did not know the exact number of AIs and humans involved. Evaluation was based on text structure, language and grammar, referencing style and quality, and relevance and validity of content as well as comprehensiveness. For each item, a value from very good (1) to very poor (5) was assigned and the overall score calculated as overall mean. An estimation of whether the text was written by a human or AI was requested. Further evaluation was performed concerning formal requirements as given in Table 3, as well as quality of references (listing in PubMed, correct citation format) and readability according to indices given in Table 5. The latter were calculated using the “Character Calculator” [20] and WebFX [21]. All texts were evaluated by PlagScan to assess percentage of resemblance to already published texts. All texts can be made accessible upon request.

**Table 2:** Score sheet for external referees based on the German grading system (1 = very good, 5 = very poor)

	1	2	3	4	5
	Very good	Good	Sufficient	Poor	Very poor
Structure					
Language					
Grammar					
Quality of references					
Referencing					
Relevance of content					
Validity of content					
Comprehensiveness					
Overall score					
Written by AI or human?					

Overall score was calculated as mean value of the above parameters and an evaluation of AI or human origin requested

**Table 3:** Overview of formal requirements concerning given limit of text length (3 pages = 1 500 words), references and text structure

AI-generator	No. of pages	References	Structure
ChatGPT-3.5	2.3	**yes**	**yes**
ChatGPT-4	1.6	**yes**	**yes**
AI-Writer	2.3	**yes**	no
Perplexity	1.3	**yes**	no
Writesonic ChatSonic	1.4	**yes**	no
Writesonic AI Article Writer 3.0	1.8	no	**yes**
Neuroflash	1.1	**yes**	**yes**
JasperChat	1.4	no	**yes**
Jasper Document editor	3.2	**yes**	no
Notion	1.2	**yes**	**yes**
Surgeon 1	1.4	**yes**	no
Surgeon 2	2.4	**yes**	**yes**
Surgeon 3	3.2	**yes**	**yes**
Surgeon 4	1.5	**yes**	no

### Data analysis

The collected data was collected in tables, analyzed for mean and standard deviation with Microsoft Excel (version 2010, Microsoft Corp., Redmond, WA USA). Inter-rater reliability was calculated using Krippendorff’s alpha and rater-to-rater reliability using Cohen’s kappa.

## Results

### Evaluation of formal requirements for scientific text

Only two texts exceeded the given length of 3 pages in Microsoft word: the AI-generated text by Jasper Document editor and the human written text by surgeon number three. Whilst all surgeons gave references, two AI-text generators did not, even though the prompt required this. Though no specification was given concerning text structure, half of the human texts and 60% of AI-generated text were structured with subheadings.

### Scoring by experienced hepatobiliary surgeons

Mean scoring of the three surgeons gave a score of 2.64 ± 0.57 for AI-generated and 1.87 ± 0.56 for human written text, according to the German school grading system (see Table 4). With 1.0 signifying “very good” and 5.0 “very poor” based on the applied gradings system, the lower score for human written text implies higher quality texts. AI-generated text was correctly identified in 73% of cases, human text in 92% of cases. Text generated by ChatGPT version 4.0 was identified as human by all surgeons, one surgeon each identified the text generated by ChatGPT-3.5, Perplexity, Writesonic’s Chatsonic, Neuroflash and Jasper Document editor as human. Only one human written text was identified as AI-generated by one surgeon. Interrater reliability as calculated via Krippendorff’s Alpha was 0.79 overall, indicating an acceptable to good reliability. When rating reliability in between raters using Cohen’s kappa, the first surgeon’s ratings were comparable to both of the other surgeons’ ratings (0.72 when compared to surgeon two and 0.68 to surgeon three). Deviation between the second and third surgeon lay at kappa = 0.54.

**Table 4:** Overview of mean expert scoring for human written and AI-generated texts, as well as texts considered human versus text considered AI

Mean scores	Human	AI	Text considered human	Text considered AI
Structure	1.8 ± 0.8	2.0 ± 1.0	1.7 ± 0.7	2.1 ± 1.0
Language	1.8 ± 0.4	2.2 ± 0.5	1.9 ± 0.3	2.3 ± 0.5
Grammar	2.0 ± 0.6	2.0 ± 0.4	1.9 ± 0.6	2.0 ± 0.4
Quality of references	2.2 ± 0.7	3.4 ± 1.2	2.2 ± 0.8	3.7 ± 1.1
Referencing	2.0 ± 0.8	3.3 ± 1.2	2.1 ± 0.7	3.5 ± 1.2
Relevance of content	1.8 ± 0.7	2.8 ± 0.9	1.9 ± 0.8	3.0 ± 0.9
Validity of content	1.8 ± 0.6	2.6 ± 0.8	1.8 ± 0.7	2.8 ± 0.8
Comprehensiveness	2.0 ± 0.8	2.8 ± 0.7	2.1 ± 0.7	3.0 ± 0.7
Overall score	1.9 ± 0.6	2.6 ± 0.6	2.0 ± 0.5	2.8 ± 0.5

Scoring analogue German marking system: 1 = very good, 5 = very poor

Therefore, we analyzed those texts which were considered to be written by humans separately and compared these to those correctly considered AI-generated as shown in Table 5: Those texts which were considered human by at least one surgeon received a better score with a mean of 1.96 ± 0.53, than those considered AI, which received a score of 2.80 ± 0.50. Overall, text considered human received better marks for structure and language, but not for grammar. Referencing, content relevance and validity as well as comprehensiveness also received better marks for human identified text.

**Table 5:** Overview on evaluation of text quality for all AI-generated and human written texts

Mean values	Human text	SD	AI text	SD	Text considered human	SD	Text considered AI	SD
FRE	26.2	5.1	17.7	6.1	20.5	6.6	19.3	7.7
FKGL	14.7	1.2	16.2	1.2	15.8	1.4	15.8	1.4
GFI	18.4	1.5	21.1	2.8	19.5	1.4	22.4	4
SMOG grade	13.3	1.1	14.7	0.7	14.3	1.1	14.6	1
CLI	15.8	1.0	17.3	1.1	16.7	1.2	17.3	1.4
ARI	14.0	2.0	16.0	1.5	15.4	1.9	15.6	1.8
Number of sentences	53.8	20.2	40.0	13.8	45.6	18.9	39.8	10.4
Number of words	1054.0	366.1	873.9	315.6	960.6	370.8	837.3	227.6
Number of complex words	284.5	103.7	261.2	93.4	274.2	105.7	252	68.5
% complex words	26.8	2.2	30.0	2.0	28.7	2.5	30.2	2.4
average words/sentence	20.2	3.9	21.9	1.6	21.5	2.9	21.1	1.5
average syllables/word	1.9	0.1	2.0	0.1	1.9	0.1	2	0.1
% Pubmed-listed references	100.0	0.0	46.0	34.4	66.7	37.6	48.2	35.5
Score (x/16)	12.8	0.3	4.9	3.8	8.1	4.7	4.8	4.2
Surgeon Score	1.87	0.56	2.64	0.57	1.96	0.53	2.80	0.50
correct AI/non-AI	0.92	0.12	0.73	0.05	-	-	-	-
PlagScan %	6.4	1.3	7.6	4.5	7.5	4	6.7	3.5

Readability indices as calculated by WebFX: FRE = Flesh Reading Ease Score, FKGL = Flesh Kinkaid Grade Level, GFI = Gunning Fogg Index, SMOG = Simple Measure of Gobbledygook, CLI = Coleman-Liau Index, ARI = Automated Readability Index, SD – standard deviation. References were scored based on listing in PubMed, existence of authors, title, publication date, journal, page-number, correct format, correct DOI.Scoring by surgical specialists was performed according to score sheet given in Table 2. Evaluation by PlagScan is given as percentage of marked words as compared to the whole text. For the right half of the table, all texts considered human by at least one surgeon are compared to those correctly considered AI-generated

### Readability and text complexity

Mean readability as measured with FRE for all AI generated texts was 8.5 points lower than for human written text as shown in Table 5 (17.7 ± 6.1 versus 26.2 ± 5.1, see Table 5). Both lie in the same range of 10–30 points, indicating a college graduate reading level. Human written text, however, is easier to read with some texts reaching the 30–50 point-range indicating college reading level. When comparing text considered human versus text considered AI-generated, this difference disappeared (20.5 ± 6.6 versus 19.3 ± 7.7). The mean number of words was lower in AI-generated text (873.9 ± 315.6) than in human written text (1054 ± 366.1). The wordcount was even lower in AI-generated text considered AI-generated (837.3 ± 227.6). Percentage of complex words lay at 30% for AI-generated text overall, and at 26.8% in human written text. Average words per sentence and syllables per word were comparable in AI-generated and human texts.

### Quality of referencing and PlagScan evaluation

Whereas 100% of references in human written text were listed in PubMed, only 46.0% of those given in AI-generated text could be verified. This rate was slightly better for those texts considered human by the experienced surgeons with 66.7% versus 48.2% for those which were correctly considered AI. In our own reference-scoring system focusing on listing in PubMed, existence and correctness of authors, title, publication date, journal, page-number, DOI, and correct referencing format, human written texts received a mean of 12.8 out of 16 points. AI-generated text only received 4.9 points overall, and 8.1 versus 4.8 for text considered human or not, respectively.

PlagScan marked 6.4% of words as suspicious in human written text, versus 7.6% in AI-generated text overall and 7.5% in text considered human versus 6.7 in text AI-generated.

## Discussion

Both AI-generated and human-written texts largely met formal requirements, with minor deviations in word count. Subheadings were used in 60% of AI and 50% of human texts, with absence linked to lack of author experience. Structural organization and referencing varied across AI systems, reflecting differing purposes the systems were designed for, such as chat-based assistance versus document creation.

AI-generated texts were misclassified as human in 30% of evaluations, while 8.3% of human texts were mistaken for AI, indicating an overlap in perceived quality. Attribution was influenced more by structure, language, and comprehensiveness than by length, readability, or lexical complexity. Referencing quality was a key differentiator: AI texts identified as AI showed poorer citation accuracy and lower inclusion of verifiable sources, whereas human texts consistently cited indexed references despite minor formatting issues. Plagiarism levels were negligible (< 10%) across all texts and primarily involved technical terminology.

Our findings align with those of Gao et al. (2023), who found that humans only identified 68% of AI-generated abstracts as AI [22] (73.3% in our study), but 14% of original, human written abstracts as being AI-generated (8.3% in our study). In June 2024, Meyer-Szary et al. published a similar experiment to ours, but went one step further [23]: They published an article fully written by Open AI’s ChatGPT-3.5, albeit prompted, fact-checked and edited by human researchers in the “Cardiology Journal” [24]. Considering the readiness for application of AI in scientific writing, the author’s own assessment of their experiment yielded a confident “not yet”. In our study we did not engage in prompt design, as we wanted to evaluate the status of text generation possibilities with the same preconditions for different AI-tools as well as humans. We expect that some AI generators could have been prompted to higher quality texts. Interestingly though, AI tools designed for text generation and document editing did not per se fare better in text evaluation that those designed as chat tools. Of the 5 chat-based assistants, 4 mislead at least one human referee into believing a human had written the text, whereas only two content creation platforms were successful in the same way. The reason for this unexpected difference might be found in the underlying LLMs: ChatGPT, Perplexity and Writesonic’s Chatsonic use either their own continuously adapted LLMs (GPT), recent versions of partly open source LLMs (Qwen) or a multi-modal approach. Most content creations platforms rely on older versions of broadly used LLM (mostly GPT).

This experiment further highlights the underlying, determining question what kind of text can be considered a scientific achievement. This is not only an ethical question, but also extends to authorship, intellectual property, and definition of scientific publications. The latter significantly influences the public understanding of science and may lead to a loss in trust of the general public in the scientific community. In this context, prompt design and human revision and quality control need to be discussed in regard to consideration as scientific achievement. We believe that especially comprehensive and thorough literature reviews on current scientific topics could potentially be a realistic application of AI in the future. An AI perusing all published content on the internet will be more successful than any human in gathering all publications on a specific matter, specifically in decidedly shorter time. However, all texts generated by AI need to be fact-checked as hallucinations still occur.

Whilst AI-generation and simplification of text open up a multitude of possible applications in medical communication to patients and/or trainees [25, 26] ranging from information material to medically trained chatbots, scientific publication using AI-text generation is seen more critical as this opens up new challenges in the fight against scientific misconduct.

Some journals and authors try to deal with these challenges by issuing guidelines or checklists. For example, Kozak (2024) gives a checklist for authors concerning the use of AI for writing and submitting manuscripts as well as a review on which recommendations some relevant committees and councils give concerning AI-use in scientific publication [27]. All exclude AI as being listed as author and refer full responsibility for AI generated content onto the human researcher. Committees agree on the necessity of full disclosure of the exact use of AI in manuscript production. For use of AI in review processes, the Committee on Publication Ethics (COP) only states that “human judgment and suitable software” should be used. The International Committee of Medical Journal Editors (ICMJE) is decidedly stricter, emphasizing the responsibility of reviewers towards authors and journals and the fact that use of AI for manuscript processing may violate confidentiality.

Nature already released ground rules for the use of AI in January 2023 [8], triggered by the release of Open AI’s ChatGPT to the general public in November 2022 and the “explosion of fun and sometimes frightening writing experiments”. These rules made clear that Nature will not accept LLM tools as credited authors on research papers due to accountability for the published text. Furthermore, it stated that use of LLM tools should be documented in the methods or acknowledgement sections. These ground rules conclude by setting the principles on which science progress needs to rely: method transparency, integrity and truth.

The JAMA Network also gives a guidance on reporting of AI use [28]. This emphasizes on the need for full transparency and accountability on use of AI for writing and publication. Especially complete naming of the AI platform and version used, as well as date of usage and description of how and to which extent the AI was utilized need to be reported. Hereby, authors need to take full responsibility for the integrity of generated content. The guideline also highlights crucial information which needs to be given for cases of use in different sections of a manuscript, especially considering potential AI-related bias.

Apart from these restrictions, some authors also find possible benefits in the use of AI, for example in the drafting of systematic reviews. Van Dijk et al. pointed out that AI may accelerate the process due to increasing number of medical-scientific publications [29]. However, they accentuated that the AI used must be specifically trained on medical publications and needs to be checked by human researchers for relevance. Wang et al. [30] even consider AI-driven plagiarism detectors as imperative in scientific writing as they may enhance the quality of scientific writing. Admittedly, this sounds promising before the backdrop of continuous challenge of misconduct in scientific writing. However, in the light of exceedingly easier (undetected) use of AI-text generators it seems to us more relevant to ensure transparency and integrity (see Nature’s ground rules) of scientific publications. Therefore, we would rather underline the need for reliable detection of AI-generated text than concentrate on the use of AI in the detection of plagiarism. One could even postulate that in a time of easily accessible AI-text generators, plagiarism is far more laborious than prompting an LLM into writing one’s manuscript.

Before this backdrop as well as considering the implications of AI in generation of opinions or decisions in the medical context, we believe that AI should be restricted to an assistant and supporting function [31]. Especially psychotherapy has experienced a massive increase in AI chatbots used for self-treatment by the population as well as development of medical app-based solutions. Whilst the general populations see their use mostly neutral (men slightly more positive than women) [32], their application is seen as critical by many professionals [33]. Some chatbot apps designed specifically for psychological support are considered to “foster emotional dependence and compromise ethical principles” [33]. This is partly due to commercial incentives, distraction by gamification or complex privacy policies, which erode the principles of autonomy, veracity, fidelity and respect as well as the underlying medical ethic of beneficence and nonmaleficence for the user.

Considering the broader picture of medicine and science, we agree with Monteith et al. [34] that limitations in the application of AI in medical decisions as well as therapeutic applications need to be critically reviewed continuously. Otherwise, analytical and therapeutic skills will be lost, which are still needed to ensure AI applications assist more than they harm. We therefore believe, that at this point, it is still partly a fantasy to let an AI text generator compile a review on its own without human revision. However, AI-generated reviews and summaries have a great potential to surpass human writing regarding comprehensiveness and actuality especially considering the time needed for a human brain to draft a thorough review on a current topic.

### Limitations of the study

The small numbers of AI-generated and human written texts, as well as only three experts who scored the texts limit the possibility of analysis to mere descriptive statistics. Furthermore, AI-text generators develop extremely fast, implying that some platforms may already have surpassed the text writing and referencing qualities found in this study.

## Conclusion

Overall, AI-text generators could already mislead experienced scientific surgeons in 26.7% of cases into believing the evaluated text to be of human origin. However, formal requirements, especially considering referencing, but also content validity and relevance are still in great need of improvement with only one of the AI-generated articles being able to fulfill quality requirements. Currently, AI should be limited to assistance and preliminary work under constant human scrutiny and control. Furthermore, in order to realistically utilize AI in scientific writing, we consider specific training on scientific texts under human control as well as continuous updating on the latest literature as essential. The responsibility for every scientific text – whether of human origin or AI-generated – needs to stay with the publishing human researcher.

## Data Availability

All data supporting the findings of this study are available within the paper.

## References

[1] Dahiya DS, Ali H, Moond V, Shah MDA, Santana C, Ali N, Sheikh AB, Nadeem MA, Munir A, Quazi MA, Bharadwaj HR, Sohail AH (2025) Large Language Models in Gastroenterology and Gastrointestinal Surgery: A New Frontier in Patient Communication and Education. Gastroenterol Res 18(2):39–4840322195. 10.14740/gr2011 10.14740/gr2011 PMC12045793 40322195

[2] Hellström T (2024) AI and its consequences for the written word. Front Artif Intell 6:132616638239498. 10.3389/frai.2023.1326166 10.3389/frai.2023.1326166 PMC10794589 38239498

[3] Guleria A, Krishan K, Sharma V, Kanchan T. ChatGPT: ethical concerns and challenges in academics and research. J Infect Dev Ctries. 2023;17(9):1292–129937824352. 10.3855/jidc.18738 10.3855/jidc.18738 37824352

[4] Benítez TM, Xu Y, Boudreau JD, Kow AWC, Bello F, Van Phuoc L, Wang X, Sun X, Leung GK, Lan Y, Wang Y, Cheng D, Tham Y, Wong TY, Chung KC. Harnessing the potential of large language models in medical education: promise and pitfalls. J Am Med Inform Assoc. 2024;31(3):776–783 Volltext: 10.1093/jamia/ocad252 10.1093/jamia/ocad252 PMC10873781 38269644

[5] Akinci D, Stanzione A, Bluethgen C, Vernuccio F, Ugga L, Klontzas ME, Cuocolo R, Cannella R, Koçak B (2024) Large language models in radiology: fundamentals, applications, ethical considerations, risks, and future directions. Diagn Interv Radiol 30(2):80–9037789676. 10.4274/dir.2023.232417 37789676 10.4274/dir.2023.232417 PMC10916534

[6] Zangrossi P, Martini M, Guerrini F, DE Bonis P, Spena G (2024) Large language model, AI and scientific research: why ChatGPT is only the beginning. J Neurosurg Sci 68(2):216–22438261307. 10.23736/s0390-5616.23.06171-4 38261307 10.23736/S0390-5616.23.06171-4

[7] Salimi A, Saheb H (2023) Large Language Models in Ophthalmology Scientific Writing: Ethical Considerations Blurred Lines or Not at All? Am J Ophthalmol 254:177–18137348667. 10.1016/j.ajo.2023.06.004 37348667 10.1016/j.ajo.2023.06.004

[8] Anonymous Tools such (2023) as ChatGPT threaten transparent science; here are our ground rules for their use. Nature 613(7945):612–136694020. 10.1038/d41586-023-00191-1 36694020 10.1038/d41586-023-00191-1

[9] Gupta R, Herzog I, Weisberger J, Chao J, Chaiyasate K, Lee ES (2023) Utilization of ChatGPT for Plastic Surgery Research: Friend or Foe? J Plast Reconstr Aesthetic Surg 80:145–147. 10.1016/j.bjps.2023.03.004 10.1016/j.bjps.2023.03.004 37023599

[10] Gupta R, Park JB, Bisht C, Herzog I, Weisberger J, Chao J, Chaiyasate K, Lee ES (2023) Expanding Cosmetic Plastic Surgery Research With ChatGPT. Aesthet Surg J 43(8):930–937. 10.1093/asj/sjad069 36943815 10.1093/asj/sjad069

[11] Sharma SC, Ramchandani JP, Thakker A, Lahiri A. ChatGPT in plastic and reconstructive surgery. Indian J Plast Surg. 2023;56(04):320–325. 10.1055/s-0043-1771514 10.1055/s-0043-1771514 PMC10497341 37705820

[12] Mugaanyi J, Cai L, Cheng S, Lu C, Huang J (2024) Evaluation of Large Language Model Performance and Reliability for Citations and References in Scholarly Writing: Cross-Disciplinary Study. J Med Internet Res 26:e5293538578685. 10.2196/52935 10.2196/52935 PMC11031695 38578685

[13] Weaver A. Palmyra LLMs empower secure, enterprise-grade generative AI for business. https://writer.com/blog/palmyra/

[14] AI-Writer com. Pricing. https://ai-writer.com/#page_pricing

[15] perplexity. Technical FAQ. https://www.perplexity.ai/hub/technical-faq

[16] Writesonic Frequently Asked Questions. https://writesonic.com/ai-article-writer-generator

[17] Neuroflash Any questions? https://neuroflash.com/neuroflash-for-marketing-teams-businesses/

[18] Anonymous AI Solutions for every kind of marketer. https://www.jasper.ai/solutions

[19] Anonymous Notion KI -Sicherheits- und Datenschutzverfahren. https://www.notion.so/de-de/help/notion-ai-security-practices

[20] Anonymous Character Counter https://charactercalculator.com/

[21] Anonymous Readability Test https://www.webfx.com/tools/read-able/

[22] Gao CA, Howard FM, Markov NS, Dyer EC, Ramesh S, Luo Y, Pearson AT (2023) Comparing scientific abstracts generated by ChatGPT to real abstracts with detectors and blinded human reviewers. NPJ Digit Med 6(1):75–637100871. 10.1038/s41746-023-00819-6 37100871 10.1038/s41746-023-00819-6 PMC10133283

[23] Meyer-Szary J, Jaguszewski M, Mikulski S (2024) Scientific writing at the dawn of AI. Cardiol J 31(3):369–37338940258. 10.5603/cj.94335 38940258 10.5603/cj.94335 PMC11229795

[24] Meyer-Szary J, Mikulski S (2024) Current understanding of Duchenne muscular dystrophy - a purported interview with a purported expert. Cardiol J 31(3):367–36838940257. 10.5603/cj.94330 38940257 10.5603/cj.94330 PMC11229802

[25] Khera R, Butte AJ, Berkwits M, Hswen Y, Flanagin A, Park H, Curfman G, Bibbins-Domingo K (2023) AI in Medicine-JAMA’s Focus on Clinical Outcomes, Patient-Centered Care, Quality, and Equity. JAMA 330(9):818–82037566406. 10.1001/jama.2023.15481 37566406 10.1001/jama.2023.15481

[26] DeTemple DE, Meine TC (2025) Comparison of the readability of ChatGPT and Bard in medical communication: a meta-analysis. BMC Med Inf Decis Mak 25(1):325. 10.1186/s12911-025-03035-2 10.1186/s12911-025-03035-2 PMC12403948 40890707

[27] Kocak Z (2024) Publication Ethics in the Era of Artificial Intelligence. J Korean Med Sci 39(33):e24939189714. 10.3346/jkms.2024.39.e249 10.3346/jkms.2024.39.e249 PMC11347185 39189714

[28] Flanagin A, Pirracchio R, Khera R, Berkwits M, Hswen Y, Bibbins-Domingo K (2024) Reporting Use of AI in Research and Scholarly Publication-JAMA Network Guidance. JAMA 331(13):1096–109838451540. 10.1001/jama.2024.3471 38451540 10.1001/jama.2024.3471

[29] van Dijk SHB, Brusse-Keizer MGJ, Bucsán CC, van der Palen J, Doggen CJM, Lenferink A (2023) Artificial intelligence in systematic reviews: promising when appropriately used. BMJ Open 13(7):e072254–07225437419641. 10.1136/bmjopen-2023-072254 37419641 10.1136/bmjopen-2023-072254 PMC10335470

[30] Wang C, Fang Y, Wang R (2024) Advancing integrity in science: the imperative for AI-driven plagiarism detection in scientific writing. Int J Surg 110(7):4488–448939042078. 10.1097/js9.0000000000001412 39042078 10.1097/JS9.0000000000001412 PMC11254215

[31] Weissman GE (2025) Evaluation and Regulation of Artificial Intelligence Medical Devices for Clinical Decision Support. Annu Rev Biomed Data Sci 8(1):81–99. 10.1146/annurev-biodatasci-103123-095824 39971383 10.1146/annurev-biodatasci-103123-095824 PMC12339208

[32] Nagel J, Hollmann K, Alt AK, Renner TJ, Conzelmann A (2026) Attitudes toward artificial intelligence and its application in psychotherapy: Assessment in healthy adults and validation of an assessment measure. Digit Health 12:20552076251410978. 10.1177/20552076251410978 41836625 10.1177/20552076251410978 PMC12982859

[33] Moylan K, Doherty K (2025) Expert and Interdisciplinary Analysis of AI-Driven Chatbots for Mental Health Support: Mixed Methods Study. J Med Internet Res 27:e67114. 10.2196/67114 40279575 10.2196/67114 PMC12064976

[34] Monteith S, Glenn T, Geddes JR, Whybrow PC, Achtyes ED, Bauer R, Bauer M. Artificial intelligence and deskilling in medicine. Br J Psychiatr. 2026 8:1–3. 10.1192/bjp.2025.10496 10.1192/bjp.2025.10496 41502298

